# Mastery-oriented and feedback-sensitive emotion profiles among written-translation MTI students in southeastern China: a Q methodology study

**DOI:** 10.3389/fpsyg.2026.1906693

**Published:** 2026-07-28

**Authors:** Ying Ye, Xinrui Wu, Kexin Weng, Rou Lin

**Affiliations:** School of Foreign Studies, Fuzhou University, Fuzhou, China

**Keywords:** achievement emotions, control-value theory, foreign language anxiety, foreign language enjoyment, foreign language learning boredom, master of translation and interpreting, Q methodology, translation learning

## Abstract

**Introduction:**

Achievement emotions have received growing attention in second language research, yet little is known about how multiple emotions are subjectively organized in professionally oriented translation learning. This study investigated enjoyment, anxiety, and boredom among written-translation students in Master of Translation and Interpreting (MTI) programs in southeastern China, examining how these emotions were subjectively organized into shared emotion profiles and how the profiles could be interpreted through control-value theory.

**Methods:**

Thirty students from two universities completed a Q-sort of 36 statements describing emotional experiences in translation learning, followed by post-sort comments. The Q-sorts were analyzed using principal component analysis and varimax rotation.

**Results:**

A two-factor solution was retained, accounting for 47% of the total variance. Factor 1 was interpreted as a mastery-oriented profile, characterized by achievement enjoyment associated with meeting standards, developing personal expression, applying translation learning to practice, and sustaining engagement in demanding tasks. Factor 2 was interpreted as a feedback-sensitive profile, in which enjoyment was closely connected with task relevance, teacher feedback, classroom atmosphere, and instructional support, while anxiety became more salient under difficult or evaluative conditions. Within the retained two-factor solution, boredom appeared less profile-defining than enjoyment and anxiety, functioning more as a situational or conditional emotion.

**Discussion:**

The findings extend research on second language achievement emotions to the context of written-translation MTI training by revealing distinct ways in which multiple emotions are subjectively organized in a professionally oriented learning context. They also demonstrate the interpretive value of control-value theory for understanding these shared emotion profiles and highlight the need for profile-sensitive translation teaching that supports both perceived control and task value.

## 1 Introduction

Emotions have become an important focus in second and foreign language learning research. Earlier studies were largely concerned with negative emotions, especially foreign language classroom anxiety (FLCA), which has been widely examined as a language-specific affective factor associated with L2 learning and achievement (Horwitz et al., 1986; Teimouri et al., 2019). With the development of positive psychology in applied linguistics, increasing attention has been paid to positive emotions, particularly foreign language enjoyment (FLE), and to the coexistence of positive and negative emotions in language classrooms (Dewaele and MacIntyre, 2014; MacIntyre and Gregersen, 2012). More recently, foreign language learning boredom (FLLB) has also been conceptualized and measured more systematically as an important achievement emotion (Li, 2021; Li et al., 2023). These developments have encouraged researchers to examine enjoyment, anxiety, and boredom not as isolated affective reactions, but as interrelated emotions that jointly shape language-learning experiences and outcomes.

Translation learning provides a relevant yet underexplored context for extending this line of inquiry. Closely related to foreign language learning but distinct from general EFL instruction, translation learning requires bilingual transfer, cross-cultural judgment, revision, professional norm awareness, and tolerance of alternative solutions, which make it emotionally intensive. Existing studies in translation and interpreting education have begun to examine emotions such as boredom, anxiety, and enjoyment, as well as learners’ affective responses to translation technologies (Hao, 2023; Hu et al., 2025; Xu and Liu, 2023; Zhang, 2022). However, much of this work has focused on specific emotions, affective predictors, or variable-centered relationships. Less is known about how translation learners subjectively organize multiple achievement emotions into shared patterns of experience, especially among students in China’s Master of Translation and Interpreting (MTI) programs.

Q methodology, a subjectivity-oriented approach, is well suited to address this gap because it combines systematic statement sorting with by-person factor analysis to identify shared viewpoints among participants (Brown, 1980; Watts and Stenner, 2012). Rather than measuring the strength of predefined variables across participants, Q methodology reveals how they prioritize, relate, and organize different aspects of their experiences. It has recently been used in applied linguistics to explore emotion-related topics such as FLE, FLLB, and classroom emotional dynamics (Fraschini, 2023; Kruk et al., 2022; Thumvichit, 2024; Wang et al., 2024). However, its application to translation learning, particularly to MTI students’ achievement emotions, is still rare.

The present study therefore uses Q methodology to explore the subjective emotion profiles of written-translation MTI students at two universities in southeastern China. It focuses on enjoyment, anxiety, and boredom, three achievement emotions that have received growing attention in EFL research but remain insufficiently examined together in translation learning. In addition, the study draws on control-value theory to interpret how the identified profiles may reflect students’ appraisals of control and value in open-ended and professionally oriented translation tasks. By doing so, it aims to extend research on L2 achievement emotions to the specialized context of MTI translation learning and to provide a subjectivity-based understanding of how translation learners experience and organize multiple emotions.

## 2 Literature review

### 2.1 Achievement emotions in EFL and translation learning contexts

Research on emotions in second and foreign language learning has moved from a predominant concern with anxiety to a broader examination of multiple achievement emotions. FLCA has long been treated as a language-specific affective construct, and its generally negative association with L2 achievement has been confirmed (Teimouri et al., 2019). By contrast, the rise of positive psychology in applied linguistics has foregrounded the role of facilitative emotions, particularly FLE, which is associated with challenge, perceived competence, supportive classroom relationships, and a sense of accomplishment (Dewaele and MacIntyre, 2014; Botes et al., 2022). More recently, as FLLB has been conceptualized and measured more systematically, studies have increasingly examined enjoyment, anxiety, and boredom as interrelated achievement emotions rather than as separate affective reactions (Li and Wei, 2023).

Across EFL and wider foreign language contexts, these three emotions are generally found to be significantly associated. Enjoyment tends to correlate negatively with both anxiety and boredom, whereas anxiety and boredom tend to be positively related (Dewaele et al., 2023; Li and Wei, 2023; Wang and Li, 2022). However, the role of each emotion varies across learning contexts, outcome measures, and learner groups. For instance, Dewaele et al. (2023) found that although FLE, FLCA, and FLLB were mutually associated, only FLCA had a direct negative effect on academic achievement. Studies in Chinese EFL contexts have also shown that enjoyment, anxiety, and boredom may contribute differently to test scores, self-perceived learning outcomes, engagement, or domain-specific language performance (Li and Han, 2022; Wang and Li, 2022). These findings suggest that L2 learners’ emotional experiences should be understood as context-sensitive configurations rather than as isolated variables with fixed effects.

Meanwhile, existing research has begun to capture the emotional specificity of translation and interpreting learning. Farias Córdova and Wiesse Ramos (2021) showed that in translation learning, students’ emotion regulation was often triggered by disappointing grades, a lack of confidence in their conceptual and thematic knowledge, course methodology, and teacher–peer relationships. Zhang (2022) identified task-based boredom and teacher-related boredom as the two major sources of boredom in Chinese translation classes, with monotonous materials, teacher-centered methods, lack of classroom activity, and insufficient feedback emerging as salient factors.

Adjacent evidence from interpreting education further suggests that bilingual transfer training is emotionally intensive. Xu and Liu (2023) found that interpreting classroom anxiety, enjoyment, and self-efficacy were significantly related to students’ self-rated interpreting competence. Hu et al. (2025), focusing on postgraduate interpreting classes, found that boredom negatively affected self-perceived interpreting learning achievement, with engagement serving as a key mediator. Technology-related learning has also entered this emotional ecology: students’ narratives about translation memory systems reveal both positive and negative emotional responses to translation technologies (Hao, 2023), while technology-based interpreting practice has been found to reduce interpreting anxiety, increase enjoyment and self-efficacy, and improve performance (Liu, 2024).

Overall, research on the interplay of enjoyment, anxiety, and boredom has developed considerably in general EFL learning contexts, but comparable work in translation learning has been scarce. Existing studies in translation and interpreting education have offered valuable insights into specific emotions and affective predictors, yet many rely primarily on questionnaire-based, variable-centered designs. What remains underexplored is a subjectivity-based account of how MTI students relate different emotional experiences to translation tasks, feedback, assessment, professional expectations, and technological change.

### 2.2 Control-value theory and EFL achievement emotions

Control-value theory (CVT) provides a useful explanatory framework for understanding why learners experience different emotions in achievement settings. According to CVT, achievement emotions are shaped primarily by learners’ appraisals of control and value. Control appraisals concern the extent to which learners believe they can manage learning activities and outcomes, while value appraisals refer to the perceived importance, usefulness, or intrinsic worth of these activities and outcomes (Pekrun, 2006). In this framework, enjoyment is likely to arise when learners perceive both sufficient control and positive value in a learning activity, whereas anxiety tends to emerge when valued outcomes are perceived as important but insufficiently controllable (Pekrun, 2006). Boredom, in turn, is typically associated with low value and may also arise when task demands are either too low or too high relative to learners’ perceived control (Pekrun and Goetz, 2024).

In EFL research, CVT has increasingly been used to explain the antecedents and consequences of language-learning emotions. Shao et al. (2020a,b) showed that Chinese university students’ perceived control and value were positively related to positive achievement emotions and foreign language performance, but negatively related to negative emotions such as anxiety, shame, hopelessness, and boredom. Focusing specifically on boredom, Li (2021) demonstrated that control-value appraisals predicted Chinese university students’ boredom in English classes both independently and interactively. More recent studies have attempted to examine multiple emotions within the same theoretical frame. Li et al. (2025), for instance, investigated enjoyment, boredom, and anxiety together with control-value appraisals, further supporting the relevance of CVT for understanding how different achievement emotions co-exist in foreign language learning. Taken together, these studies suggest that control-value appraisals help explain not only the intensity of individual emotions but also the ways in which enjoyment, anxiety, and boredom may co-exist in foreign language learning. These findings make CVT especially valuable for interpreting the context-sensitive nature of EFL emotions.

Despite its growing use in EFL emotion research, CVT has only recently begun to inform studies of achievement emotions in translation learning. Translation classrooms are theoretically well suited to a control-value perspective because they involve open-ended tasks, professional norms, and repeated negotiation between linguistic accuracy, communicative adequacy, and cultural appropriateness. One relevant study is Man’s (2025) comparison of situated expectancy-value theory (SEVT) and CVT in explaining multilingual students’ translation classroom engagement. Although this study brought control-value-related constructs into translation classroom research, it focused primarily on engagement and model comparison rather than on the subjective organization of multiple achievement emotions. Thus, existing CVT-informed research has provided important theoretical resources for understanding EFL achievement emotions, but its application to translation learning is yet to be extended. In particular, little is known about how control- and value-related meanings may help interpret shared profiles of enjoyment, anxiety, and boredom in MTI translation learning.

### 2.3 Q methodology in EFL achievement emotion research

Q methodology offers a productive way of examining learners’ subjective viewpoints in language learning. Unlike conventional survey-based approaches that primarily measure the distribution and strength of predefined variables, Q methodology is designed to reveal shared patterns of subjectivity through the systematic sorting of statements and factor analysis of participants’ viewpoints (Brown, 1980; Watts and Stenner, 2012). Recent methodological discussions in applied linguistics have emphasized that Q methodology is especially useful for exploring complex, context-dependent, and internally organized perspectives (Morea and Ghanbar, 2024). This methodological affordance helps explain its increasing application in research on emotion-related topics in foreign language learning.

In EFL emotion research, Thumvichit (2024) examined EFL learners’ subjective perspectives on FLE in online classes, while Wang et al. (2024) investigated what makes EFL learning enjoyable for Chinese tertiary-level students and identified distinct patterns of FLE related to informativeness, independent learning, collaborative work, and interestingness. In terms of boredom, Kruk et al. (2022) used Q to explore Iranian EFL learners’ subjective views of potential sources of foreign language learning boredom, showing that boredom is not a uniform classroom experience but is shaped by different combinations of task-, teacher-, peer-, and learner-related factors. Beyond single-emotion studies, Fraschini (2023) used an intensive Q-methodological single-case design to examine language learners’ emotional dynamics across classroom episodes, demonstrating that learners may respond to the same classroom events in both shared and idiosyncratic ways. More recently, Wang and Wang (2025) examined boredom, enjoyment, and emotion regulation among Chinese students majoring in dual foreign languages, further showing the value of Q for capturing how multiple affective experiences are organized in multilingual learning contexts.

However, Q methodology has not been widely used to investigate translation learning. Existing Q-based work in translation studies has mainly addressed topics such as ethical perspectives on translation technology among translation students and professionals (Li, 2025), and learning motivation among Chinese students majoring in translation and interpreting in the era of generative AI (Xing and Zhao, 2026), rather than achievement emotions in translation learning. Translation learning, especially at the MTI level, provides a highly relevant context for Q-methodological inquiry because students’ emotional experiences are shaped by open-ended translation tasks, teacher and peer evaluation, professional norms, certification pressure, and changing expectations brought about by machine translation and AI-assisted translation.

In sum, although the interplay of enjoyment, anxiety, and boredom has been increasingly examined in EFL learning, little is known about how these emotions are subjectively configured among Chinese MTI students in translation learning. Moreover, while CVT offers a useful lens for interpreting learners’ appraisals of control and value, its explanatory potential has not been sufficiently explored in this context. To address these gaps, the present study uses Q methodology to identify shared emotion profiles among Chinese MTI students and draws on CVT to interpret how control- and value-related meanings may help explain these shared emotion profiles. Specifically, this study addresses the following research questions:

RQ1: What shared emotion profiles of enjoyment, anxiety, and boredom can be identified among MTI students specializing in written translation in the present study?

RQ2: How can these profiles be interpreted through the lens of control-value theory in the context of translation learning?

## 3 Methodology

### 3.1 Research context and participants

This study was conducted in two universities in southeastern China, both of which have offered MTI programs for more than 15 years. The MTI curriculum in these programs includes compulsory translation courses and regular translation assignments, through which students are exposed to a range of classroom-based and practice-oriented translation tasks. These programs therefore provided an appropriate context for examining how translation learners experience and organize enjoyment, anxiety, and boredom in relation to translation learning.

Thirty MTI students specializing in written translation participated in the study. They were recruited through snowball sampling on a voluntary basis. Nineteen participants were first-year postgraduate students and 11 were second-year students. Twenty-six participants (86.7%) had undergraduate backgrounds in English-related majors, including English, translation, business English, and English education, while four (13.3%) came from non-English majors such as finance, Chinese language and literature, public administration, and primary education. Participants also reported varied levels of translation-related experience. About half had no prior translation internship or project experience, while the others had participated in one or more translation-related activities, such as conference translation, escort interpreting, localization, translation editing, engineering-text translation, or volunteer translation for institutional events. In relation to translation certification, most participants were preparing for translation qualification examinations such as CATTI, a national translation qualification examination in China, while several had already passed CATTI Level-II or Level-III translation tests.

The participants were suitable for the present study for three reasons. First, as MTI students specializing in written translation, they had direct and recent experience of translation courses, assignments, classroom evaluation, and certification-related assessment. Second, their learning experiences involved both classroom-based tasks and broader professional expectations, including certification preparation and awareness of technological changes in the translation industry. Third, their varied educational and practical backgrounds made it possible to capture a range of subjective viewpoints on achievement emotions in translation learning.

Before data collection, participants were informed of the purpose of the study, the voluntary nature of their participation, and their right to withdraw at any stage. They were also informed that their responses would be used only for research purposes and reported anonymously. Informed consent was obtained from all participants before they completed the Q-sort and the post-sort questionnaire. To protect participants’ privacy, institutional names and participant identifiers were anonymized in the analysis and reporting.

### 3.2 Q-set development

The development of the Q-set followed a multi-stage process. In Q methodology, the Q-set should provide a structured yet manageable representation of the range of viewpoints relevant to the research topic (Brown, 1980; Watts and Stenner, 2012). In the present study, the Q-set was designed to capture MTI students’ subjective emotional experiences in translation learning, with particular attention to the ways in which enjoyment, anxiety, and boredom may appear separately or in combination.

The initial pool of candidate statements was generated from two sources. One source was previous research on achievement emotions in EFL, translation, and interpreting learning contexts, including studies on FLE, FLCA, FLLB, translation classroom boredom, teacher feedback, assessment, and translation technology. The other source was an informal pilot interview with two MTI students specializing in written translation. The interview helped generate statements that were close to the students’ own wording and lived experiences, covering emotional responses to translation tasks, classroom activities, teacher feedback, peer interaction, assessment, certification examinations, professional identity, and translation technologies.

After the immediate removal of overlapping, overly general, or low-relevance items, a documented working pool of 49 candidate statements was retained. These statements covered six broad domains, i.e., translation tasks and texts, teachers and classroom pedagogy, peers and interaction, assessment, examinations and certificates, self-efficacy and professional identity, and workload, energy and burnout. The working pool was then reviewed and refined through several rounds of reduction. Statements that described highly similar situations were merged; items rarely mentioned in the pilot interview or only weakly related to translation-learning emotions were removed; and linguistically complex or ambiguous statements were rewritten to improve clarity and readability. During this process, attention was also paid to balancing statements representing enjoyment, anxiety, boredom, and combinations of these emotions, so that the final Q-set could capture both positive and negative achievement emotions as well as their co-occurrence in translation learning. This process was consistent with the principle that a Q-set should be broad enough to represent the relevant concourse while remaining manageable for participants during sorting (Watts and Stenner, 2012; McKeown and Thomas, 2013).

To further improve the clarity and relevance of the statements, two other MTI students specializing in written translation were invited to review the candidate statements. They were asked to identify items that seemed unclear, repetitive, or distant from their own translation-learning experience. Based on their feedback, further revisions were made to the wording and coverage of the statements. A pilot test was then conducted with three additional MTI students to check whether the statements were understandable and whether the sorting task could be completed within a reasonable time. Minor wording adjustments were made after the pilot test.

The final Q-set consisted of 36 statements covering the six domains described above and representing the major contexts in which MTI students experience achievement emotions in translation learning. Each statement was coded according to its content domain and emotional orientation, including enjoyment, anxiety, boredom, and mixed orientations. The initial coding was conducted by the first author and reviewed by another member of the research team familiar with achievement emotion research and translation pedagogy. Ambiguous cases, particularly statements involving mixed emotional orientations, were discussed until consensus was reached. The Q-set was administered in simplified Chinese, and Appendix A presents the original Chinese statements together with their English translations, content domains, and emotional orientations.

### 3.3 Q-sorting procedure

The formal Q-sorting task was conducted through Q-TIP, an online platform for administering Q sorts. Participants were asked to sort the 36 Chinese statements according to the extent to which each statement reflected their own emotional experiences in translation learning. The sorting instruction was therefore based on personal relevance rather than general agreement.

**Figure 1:**
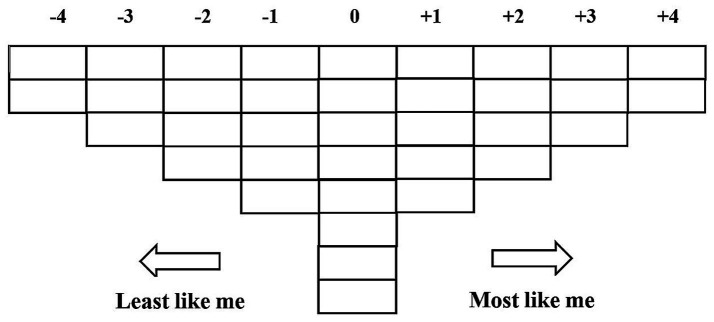
Forced distribution grid used in the Q-sorting task.

Following standard Q-sorting procedures (Brown, 1980), participants placed the statements onto a forced quasi-normal distribution grid (see Figure 1), which contained nine positions, ranging from −4 (“least like me”) to +4 (“most like me”). A nine-position grid was used in the current study because the 36-statement Q-set was relatively compact. The −4 to +4 distribution provided sufficient differentiation among statements while keeping the online sorting task manageable for participants. Participants completed the task by dragging and dropping the statements onto the distribution grid. Because Q sorting requires participants to evaluate statements relationally rather than independently, they were encouraged to consider the relative placement of the statements before submitting their final sort.

After completing the Q-sort, participants filled in a short post-sort questionnaire through *Wenjuanxing*, a Chinese online survey platform. The questionnaire collected background information, including participants’ educational background, translation-course experience, translation-related practice, preparation for translation certification examinations, and other relevant learning experiences. To support the interpretation of the Q-sort results, participants were also asked two open-ended post-sort questions. Specifically, they were invited to select the statement they felt was most representative from the two statements placed at +4 and explain why it was “most like me.” They were then asked to do the same for the statement they felt was most representative from the two statements placed at −4. Where willing, participants could also briefly explain the other statement placed at each extreme. These post-sort comments were used to contextualize and interpret the factor arrays in the Results section (Watts and Stenner, 2012).

### 3.4 Data analysis

The completed Q-sorts were exported from Q-TIP and imported into Ken-Q Analysis Desktop Edition (KADE), a software package designed for Q-methodological analysis (Banasick, 2019). In Q methodology, the analysis focuses on correlations among participants’ Q-sorts rather than correlations among variables. By-person factor analysis was therefore used to identify groups of participants who sorted the statements in similar ways and thus shared comparable viewpoints on achievement emotions in translation learning.

Principal component analysis (PCA) was used for factor extraction, followed by varimax rotation. The number of retained factors was determined by considering both statistical and interpretive criteria, including eigenvalues, the pattern of eigenvalues, explained variance, the number of defining Q-sorts, factor correlations, and the theoretical meaningfulness of the resulting factor arrays (Watts and Stenner, 2012; McKeown and Thomas, 2013). The PCA extraction yielded eight components with eigenvalues greater than 1. The eigenvalues of these components were 11.59, 2.51, 1.92, 1.72, 1.49, 1.42, 1.23, and 1.09, respectively. Because the eigenvalue-greater-than-one rule may overextract factors in exploratory analyses, eigenvalues were treated as a preliminary guide rather than as the sole retention criterion. Two-, three-, and four-factor varimax-rotated solutions were therefore inspected using the same extraction, rotation, and auto-flagging procedures.

To determine which Q-sorts could be used to define each factor, KADE’s auto-flagging function was used. Q-sorts were automatically flagged as defining sorts when they met two criteria: their factor loading exceeded the significance threshold at the *p* < 0.05 level, and the majority of their common variance was accounted for by that factor. The statistical significance threshold for factor loadings was calculated as 1.96 × 1/ $\sqrt{N}$ , where N refers to the number of statements in the Q-set (Brown, 1980; Watts and Stenner, 2012). With 36 statements in the present study, absolute loadings of 0.33 or higher were considered statistically significant at the *p* < 0.05 level. The retained two-factor solution accounted for 47% of the total variance, with Factor 1 explaining 25% and Factor 2 explaining 22%. Fifteen Q-sorts were flagged as defining sorts for Factor 1 and fourteen for Factor 2; one Q-sort was not flagged as a defining sort. The correlation between the two factors was 0.57, indicating that the two profiles were related but distinguishable.

For each factor, KADE generated a factor array, which represents an idealized Q-sort for that factor. These factor arrays formed the main basis for interpreting the shared emotion profiles. Statements placed at the extreme ends of the arrays were examined first, as they indicated the strongest identification or disidentification within each profile. Distinguishing statements were then used to identify statements that differentiated one factor from the other, while consensus statements were used to identify shared views across the two profiles. Distinguishing statements at the *p* < 0.01 level were treated as particularly strong evidence for factor interpretation, while those at the *p* < 0.05 level were used as supplementary evidence. Participants’ post-sort comments were also consulted to contextualize the factor arrays and to support the interpretation and naming of the profiles. They were examined in relation to participants’ extreme placements and factor membership, following a crib-sheet logic in which comments on the most strongly endorsed and rejected statements were read alongside the relevant factor arrays. The comments were not analyzed as an independent qualitative dataset or used to generate separate themes. Because participants were mainly asked to comment on statements placed at +4 and −4, these comments were treated as useful but limited contextual evidence. The post-sort comments were originally written in Chinese, and the excerpts quoted in the manuscript were translated into English by the authors and checked against the original Chinese comments to preserve meaning.

In reporting the retained solution, the Results section provides a concise narrative interpretation of the main factor patterns, while Appendix B presents the full Q-methodological results. The complete statement-level results are reported in Appendix B, Table B1, which presents the factor arrays for all 36 statements, including Q-sort values, z-scores, distinguishing/consensus status, and significance levels. Participant-level factor loadings are reported in Appendix B, Table B2, with defining and non-defining sorts indicated.

## 4 Results

### 4.1 Overview of the factor solution

The retained two-factor solution accounted for 47% of the total variance. As shown in Table 1, Factor 1 explained 25% of the variance and was defined by 15 Q-sorts, while Factor 2 explained 22% of the variance and was defined by 14 Q-sorts. One Q-sort was not flagged as a defining sort. The correlation between the two factors was 0.57, suggesting that the two profiles were related but distinguishable rather than mutually opposed.

**Table 1:** Characteristics of the retained two-factor solution.

Factor	Interpreted profile	Defining Q-sorts	Explained variance	Core pattern
Factor 1	Mastery-oriented profile	15	25%	Enjoyment organized around standards, mastery, personal expression, practical application, and low self-doubt
Factor 2	Feedback-sensitive profile	14	22%	Enjoyment organized around practical value, teacher feedback, classroom climate, and instructional support
Total		29	47%	Two related but distinguishable emotion profiles

One Q-sort was not flagged as a defining sort. The correlation between Factor 1 and Factor 2 was 0.57.

To assess the robustness of this retention decision, alternative three- and four-factor varimax-rotated solutions were also inspected using the same extraction and flagging procedures. Table 2 summarizes the comparison of the retained and alternative factor solutions.

**Table 2:** Comparison of retained and alternative factor solutions.

Solution	Defining Q-sorts	Total defining Q-sorts	Rotated variance	Highest factor correlation
Two-factor solution	15, 14	29/30	47%	0.57
Three-factor solution	11, 8, 6	25/30	54%	0.57
Four-factor solution	9, 3, 2, 9	23/30	58%	0.70

All solutions were based on the extraction of eight principal components followed by varimax rotation. Defining Q-sorts were identified using the same auto-flagging criteria.

Although the three- and four-factor solutions explained more variance, they defined fewer Q-sorts and produced less parsimonious structures. In the three-factor solution, the additional factor was organized mainly around achievement, competence confirmation, personal expression, and technology-related professional unease rather than boredom. The four-factor solution produced smaller defining groups and a higher maximum factor correlation, suggesting greater fragmentation and overlap. Neither alternative solution yielded a stable boredom-centered viewpoint. The two-factor solution was therefore retained as the most coherent and parsimonious solution for the present research questions.

Table 3 summarizes the background characteristics of the defining participants for each factor, including year of study, undergraduate major, translation-related practice experience, and certification status. It indicates that both profiles included participants from different years of study, undergraduate backgrounds, practice experiences, and certification statuses.

**Table 3:** Background characteristics of defining participants by factor.

Background characteristic	Factor 1 (*n* = 15)	Factor 2 (*n* = 14)
Year 1	11	7
Year 2	4	7
English-related undergraduate major	14	11
Non-English undergraduate major	1	3
No translation practice experience	7	8
At least one translation-related practice experience	8	6
Preparing for translation certification	10	9
Passed a translation certification test	5	3
Not preparing for certification	0	2

The table includes defining Q-sorts only. One non-defining Q-sort is not included.

Drawing on the complete Q-methodological results reported in Appendix B and participants’ post-sort comments, Factor 1 was interpreted as a mastery-oriented profile, while Factor 2 was interpreted as a feedback-sensitive profile. These labels refer to shared viewpoints rather than fixed psychological types. Factor 1 foregrounded enjoyment associated with standards, personal expression, practical application, and low self-doubt, whereas Factor 2 emphasized achievement and practical value but connected enjoyment more closely with teacher feedback, classroom climate, and instructional support.

In the following sections, selected statements are discussed in relation to Q-methodological outputs. The position of each statement in the relevant factor array is reported in parentheses, with positive values indicating stronger identification with the statement and negative values indicating weaker identification. For example, S4 (+4) means that Statement 4 was placed at the most positive end of the factor array, whereas S29 (−4) means that Statement 29 was placed at the most negative end.

### 4.2 Factor 1: Mastery-oriented profile

Factor 1 was interpreted as a mastery-oriented profile. In the factor array, this profile was organized around achievement enjoyment derived from producing high-quality translations, developing personal expression, applying translation learning to practice, and sustaining engagement in demanding translation tasks. It also showed strong disidentification with self-doubt, boredom with revision, and fatigue from prolonged translation practice.

The strongest positive statements for Factor 1 were S8 (+4), which concerned the meaning and enjoyment of producing a literary translation with personal understanding and style, and S4 (+4), which described a strong sense of achievement when a translation met course requirements or professional standards and was also personally satisfactory. Factor 1 also placed high value on applying translation learning to practice (S7: +3). These placements suggest that enjoyment in this profile was closely tied to achievement, competence, personal meaning-making, and the perceived usefulness of translation learning.

The distinctiveness of this profile was further supported by statements that Factor 1 ranked higher than Factor 2. In particular, Factor 1 placed much stronger emphasis on personal interpretation and style in literary translation (S8: +4 vs. +1), immersion in translation tasks when in good form (S35: +2 vs. −3), and confidence gained from good results in translation courses, competitions, or certificate examinations (S26: +2 vs. 0). These placements indicate that students represented by this profile tended to organize their emotional experiences around mastery, challenge, refinement, and competence confirmation.

The negative end of the factor array was equally informative. Factor 1 strongly rejected the statement that repeated revision and word choice were tedious (S6: −4), and also strongly disagreed with self-doubt about suitability for translation (S29: −4). Fatigue or boredom from prolonged translation practice was also placed at the negative end (S33: −3). These placements reveal that this profile was not defined by an absence of demanding tasks, but by the tendency to experience difficulty, revision, and sustained practice as part of meaningful translation learning rather than as sources of disengagement.

Post-sort comments from defining participants were consistent with this interpretation. A defining participant for Factor 1 described a successful translation as “like creating a work of art” (P22, Factor 1). Another participant, who emphasized the practical application of translation learning, explained that learning became more meaningful when it “did not remain only in the classroom or on paper” but produced “greater value” in use (P05, Factor 1). Participants also repeatedly rejected the idea that revision was boring. One defining participant described translation as “a process of gradual improvement” (P01, Factor 1), while another explained that each satisfying revision brought a renewed sense of achievement and motivated further refinement (P27, Factor 1). These comments show that the mastery-oriented profile was characterized by a close connection between achievement, self-improvement, and sustained engagement in translation practice.

### 4.3 Factor 2: Feedback-sensitive profile

Factor 2 was interpreted as a feedback-sensitive profile. In the factor array, this profile was not characterized by low achievement value. Like Factor 1, it strongly endorsed achievement from meeting course or professional standards (S4: +4) and practical application of translation learning (S7: +4). Its distinctiveness lay in the fact that enjoyment was more closely connected with task relevance, teacher feedback, classroom climate, and instructional support, while anxiety and pressure were more salient when tasks felt difficult, insufficiently scaffolded, or embedded in evaluative situations.

The strongest positive placements in Factor 2 were S7 (+4) and S4 (+4), followed by statements concerning real-life or socially relevant texts (S2: +3), specific positive teacher feedback (S16: +3), and teachers’ humor or background storytelling in translation classes (S13: +3). These placements show that students represented by this profile experienced enjoyment when translation learning was practically meaningful and when feedback or classroom interaction made their progress visible. Compared with Factor 1, Factor 2 placed higher emphasis on real-life or socially relevant texts (S2: +3 vs. +2) and classroom atmosphere created by teacher humor and storytelling (S13: +3 vs. +1).

The profile was also marked by sensitivity to task demands and evaluative conditions. Factor 2 ranked higher than Factor 1 on pressure caused by highly difficult translation tasks (S1: +2 vs. +1), lack of domain knowledge in specialized non-literary translation (S9: +2 vs. +1), and peer comparison when classmates displayed stronger translation ability (S21: +2 vs. 0). It also placed higher than Factor 1 on frustration caused by heavy assignments with insufficient explanation (S17: +1 vs. 0), pressure from handling translation assignments alongside other coursework (S34: +1 vs. −2), public criticism by the teacher (S14: +1 vs. 0), and grade-centered teaching that neglected students’ feelings (S15: +1 vs. 0). Although these statements were not always placed at the extreme positive end, their relative positions helped distinguish this profile from Factor 1 by foregrounding the importance of instructional and evaluative support.

The negative end of the factor array further clarified the conditional nature of this profile. Factor 2 strongly rejected the statement about viewing translation as a possible long-term career with both anticipation and tension (S30: −4), and placed low the statements about immersion in translation tasks (S35: −3), stable enjoyment under reasonable course arrangements (S36: −3), and increased enjoyment of translation thinking and expression since entering the program (S28: −3). These placements suggest that Factor 2 was less organized around sustained task immersion, stable positive engagement, or long-term professional anticipation than Factor 1. Its enjoyment was more closely tied to the immediate meaningfulness of tasks and the quality of classroom support.

Post-sort comments from defining participants further clarified the support-contingent nature of this profile. Several defining participants for Factor 2 who placed real-life or socially relevant texts at the positive extreme explained that such texts made translation feel meaningful, fresh, and connected with current social life. One participant described such translation as a way to “build an information bridge” and reduce communication gaps (P04, Factor 2). Another wrote that socially relevant texts amplified the sense of achievement because they made learning feel useful and “in tune with the times” (P18, Factor 2). Comments also highlighted the role of feedback and support. One participant explained that heavy assignments without adequate explanation were frustrating because feedback was necessary for real improvement (P28, Factor 2). Another participant, who rejected immersion in translation tasks, noted that immersion occurred mainly in examination contexts, where concentration came from pressure to complete the task rather than from enjoyment of translation itself (P06, Factor 2). These comments suggest that the feedback-sensitive profile was not defined by weak achievement value. Rather, it should be understood as a task- and support-contingent configuration in which enjoyment became salient when immediate task value, visible progress, and constructive classroom conditions were present.

### 4.4 Shared patterns and the limited differentiating role of boredom

In addition to the differences between the two profiles, the consensus statements indicate that the two profiles shared a common valuation of translation learning. Both factors placed strong positive emphasis on achievement from producing a satisfactory translation that met course or professional standards (S4: +4/+4) and on the practical application of translation learning (S7: +3/+4). Both profiles also valued teachers’ specific recognition of strengths in students’ translations (S16: +3/+3) and a classroom environment that encouraged alternative translations and tolerated mistakes (S11: +3/+2). These shared placements suggest that the two profiles were not opposed in their basic valuation of translation learning. Rather, both were organized around achievement value and practical relevance, while differing in how enjoyment, anxiety, feedback, and task-related pressure were configured.

As shown in Table 4, boredom-related statements played a limited role in differentiating the two profiles. Most were placed at low or moderate positions rather than at the positive extreme of either factor array. Factor 1 strongly rejected boredom with repeated revision and fatigue from prolonged translation practice (e.g., S6, S33), while Factor 2 gave only mildly positive placements to boredom-related concerns linked to insufficient support or workload pressure (e.g., S15, S17, S34). Statements related to low interaction or ineffective group work also remained near the negative side or middle of the arrays (e.g., S10, S20). Post-sort comments further supported this pattern: participants often described revision and word choice not as tedious repetition, but as a process of improvement, self-correction, and refinement.

**Table 4:** Placement of boredom-related statements in the retained two-factor solution.

Statement	Emotional orientation	Factor 1 Q-SV	Factor 2 Q-SV
S3	Boredom + anxiety	−3	−4
S6	Boredom	−4	−2
S10	Boredom	−1	−1
S15	Boredom + anxiety	0	+1
S17	Boredom + anxiety	0	+1
S20	Boredom	−1	0
S22	Boredom	−2	0
S32	Boredom + anxiety	−1	−2
S33	Boredom	−3	−1
S34	Boredom + anxiety	−2	+1

Q-SV = Q-sort value in the factor array. Boredom-related statements include statements coded as boredom or boredom + anxiety in Appendix A.

Taken together, these results suggest that boredom was present in the Q-set but was less central than enjoyment and anxiety in differentiating the two profiles. The main contrast between Factor 1 and Factor 2 therefore lay not in whether boredom was simply present or absent, but in how achievement enjoyment, perceived competence, feedback, and task-related pressure were subjectively organized. Boredom appeared more as a situational or secondary concern, emerging under specific conditions such as low interaction, insufficient support, ineffective group work, or workload pressure, rather than as an independent emotion profile within the retained two-factor solution.

## 5 Discussion

The two-factor solution shows that the participating MTI students’ achievement emotions were organized less by single emotions than by broader appraisal patterns. The mastery-oriented profile was organized around competence, standards, personal expression, and sustained engagement, whereas the feedback-sensitive profile was more dependent on task relevance, feedback quality, classroom climate, and instructional support. The discussion below interprets this asymmetry through control-value theory, focusing first on the two profiles and then on the less differentiating role of boredom.

CVT is used here as an interpretive lens rather than as a measured explanatory model. The interpretation draws on the content of the Q-statements, factor arrays, and post-sort comments to examine how participants’ emotion profiles may be understood in relation to perceived competence, task value, feedback, standards, and task demands. Several Q-statements were especially relevant to this interpretation because they occupied salient positions in the factor arrays and concerned control-, value-, or emotion-related meanings. These statements are summarized in Table 5.

**Table 5:** Selected Q-statements informing the control-value interpretation.

Statement	Factor-array evidence	Control-related reading	Value-related reading	Focal emotion
S4	F1 + 4 / F2 + 4	competence confirmed through meeting standards	achievement and professional standards	Enjoyment
S7	F1 + 3 / F2 + 4	learning can be applied in practice	practical utility of translation learning	Enjoyment
S8	F1 + 4 / F2 + 1	agency in producing one’s own version	intrinsic and expressive value	Enjoyment
S16	F1 + 3 / F2 + 3	feedback supports perceived competence	visible progress and recognition	Enjoyment
S35	F1 + 2 / F2–3	task immersion and manageability	engagement in translation activity	Enjoyment
S1	F1 + 1 / F2 + 2	control reduced by difficult task demands	valued task completion under uncertainty	Anxiety
S21	F1 0 / F2 + 2	peer comparison threatens perceived competence	evaluative value of proficiency	Enjoyment + anxiety
S34	F1–2 / F2 + 1	workload constrains task control	competing demands across courses	Boredom + anxiety
S6	F1–4 / F2–2	revision not strongly experienced as loss of control	revision retained as meaningful improvement	Boredom
S3	F1–3 / F2–4	low challenge not central to either profile	low task value rejected as defining experience	Boredom + anxiety

CVT, control-value theory. Statements are grouped by the focal emotion most relevant to the interpretation.

### 5.1 Mastery orientation viewed through control and value appraisals

Viewed through control-value theory, the mastery-oriented profile is suggestive of relatively strong perceived competence and task value. According to control-value theory, achievement emotions are shaped primarily by learners’ appraisals of control and value. Enjoyment is likely to arise when learners perceive learning activities or outcomes as both valuable and controllable, whereas anxiety tends to emerge when valued outcomes are perceived as uncertain or insufficiently controllable (Pekrun, 2006; Pekrun and Stephens, 2010). In this study, Factor 1 combined endorsement of standards, personal expression, practical application, and immersion with rejection of self-doubt and revision-related boredom. This pattern suggests that demanding translation tasks were not primarily experienced as threats, but as meaningful opportunities for refinement, self-expression, and professional growth.

This interpretation is consistent with CVT-informed EFL research showing that control and value appraisals are closely associated with learners’ achievement emotions. Shao et al. (2020a,b), for example, found that perceived control and value were positively related to positive achievement emotions and language performance, but negatively related to negative emotions. More recent research has also shown that enjoyment, anxiety, and boredom are interrelated in foreign language learning and can be meaningfully examined within a control-value framework (Li and Xing, 2024; Li et al., 2025). The present study adds to this line of research by showing how these appraisal-emotion relations may be subjectively configured in translation learning. For mastery-oriented MTI students, high standards, complex texts, and repeated revision could remain compatible with enjoyment when students felt capable of improving their translations and regarded the task as personally or professionally meaningful.

The profile also resonates with research on FLE, which has linked positive language-learning experiences to challenge, perceived competence, teacher support, and a sense of accomplishment (Dewaele and MacIntyre, 2014; Dewaele et al., 2018). However, the translation-learning context gives these elements a more specialized form. Enjoyment was not simply linked to pleasant classroom interaction or general interest in language learning but was tied to the craft of producing a satisfactory translation, the possibility of expressing one’s own interpretation, and the gradual improvement of a text through revision. Post-sort comments further illustrate this point. As noted above, participants’ comments linked successful translation and revision with artistry, gradual improvement, and sustained achievement. Thus, the mastery-oriented profile should not be understood as an absence of difficulty or pressure. In effect, difficulty seemed to be experienced as controllable and worthwhile, allowing enjoyment to remain central even in demanding situations.

### 5.2 Feedback sensitivity viewed through task value and learning support

From a control-value perspective, Factor 2 can be read as a profile in which task value remained salient, while perceived control appeared more dependent on classroom conditions. Factor 2 also valued standards and practical application, but its enjoyment was more closely tied to task relevance, teacher feedback, classroom atmosphere, and instructional support. The placements suggest that feedback and classroom support may have functioned as control-supportive conditions, helping students make standards visible, recognize progress, and manage uncertainty in open-ended translation tasks.

This interpretation is supported by previous EFL and translation-learning research showing that teacher- and classroom-related factors are closely connected with learners’ emotional experiences. Studies on FLE have shown that teacher behavior, classroom atmosphere, and perceived support can shape students’ enjoyment and anxiety (Dewaele and MacIntyre, 2014; Dewaele et al., 2018). Dewaele and Li (2021) also found that perceived teacher enthusiasm was related to EFL learners’ enjoyment, boredom, and social-behavioral engagement. In translation-learning research, Zhang (2022) identified task-based and teacher-related boredom as major sources of boredom in Chinese translation classes, while Man (2025) showed that expectancy and anxiety significantly predicted multilingual students’ translation classroom engagement. Together these studies suggest that students’ emotions in language and translation learning are shaped not only by task value, but also by whether classroom conditions make learning feel manageable, supported, and emotionally safe.

The present study adds to this literature by showing how such conditions are subjectively organized in a feedback-sensitive emotion profile. For these MTI students, feedback did not function merely as external praise. It appeared to make standards visible, confirm progress, and help students judge whether they were improving in an open-ended task environment. At the same time, anxiety and pressure became more salient in situations that could undermine a sense of control, such as highly difficult texts, insufficient domain knowledge, or accumulated assignments. They were also heightened under evaluative conditions involving peer comparison, public criticism, or grade-centered teaching. This pattern suggests that feedback-sensitive students may experience translation learning as valuable but not always fully controllable. In this profile, enjoyment appeared to depend less on stable immersion or long-term professional anticipation and more on whether the immediate learning environment provided clear and supportive conditions for engaging with translation tasks.

### 5.3 The limited differentiating role of boredom

A further finding concerns the role of boredom. Although boredom was included in the Q-set as one of the three focal achievement emotions, it appeared less profile-defining than enjoyment and anxiety within the retained two-factor solution. Table 4 helps clarify this asymmetry. Boredom-related statements were generally placed at low or moderate positions, and both profiles rejected the idea that repeated revision and word choice were simply tedious. From a control-value perspective, this suggests that boredom may have been less central because translation learning retained sufficient value for most participants. Even potentially repetitive activities, such as revision, could be interpreted as meaningful when they were connected with improvement, competence development, and professional standards.

This finding should not be taken to mean that boredom was absent from translation learning. Rather, it suggests that boredom was more situational than profile-defining in the present study. Previous EFL research has shown that boredom is a significant negative emotion in language learning and is closely associated with learners’ control-value appraisals (Li, 2021; Li et al., 2023). Research on Chinese translation classes has also identified task-based and teacher-related boredom as major sources of translation classroom boredom (Zhang, 2022). The placement of boredom-related statements in Table 4 and the inspection of alternative factor solutions (see 4.1) point to the same pattern: boredom did not organize a stable shared viewpoint in this study. Instead, boredom-related concerns appeared in relation to particular tasks or classroom conditions, such as low interaction, ineffective group work, insufficient instructional support, workload, and task difficulty. These concerns were visible in the Q-set, but they did not outweigh the shared sense of achievement value and practical relevance that characterized both profiles.

The limited differentiating role of boredom may stem from the professional orientation of MTI programs. Because translation tasks are closely tied to professional identity, certification, and future careers, even potentially tedious activities such as revision may be appraised as refinement when they support competence development and professional standards. Boredom’s role therefore depends not only on task features or teaching methods, but also on how learners embed those tasks within broader value systems.

On the whole, the results suggest that enjoyment, anxiety, and boredom were organized asymmetrically in this study. Enjoyment and anxiety played a more visible role in differentiating the mastery-oriented and feedback-sensitive profiles, whereas boredom appeared as a secondary or conditional emotion. This adds nuance to current research on multiple foreign language emotions. While recent studies have shown that enjoyment, anxiety, and boredom are interrelated and can jointly shape learning outcomes (Li and Xing, 2024; Li et al., 2025), the present Q-methodological findings suggest that their subjective organization may not be equally balanced in all learning contexts. In this MTI setting, boredom was visible but did not organize a separate shared viewpoint when translation learning retained achievement and professional value.

## 6 Conclusion, implications, and limitations

### 6.1 Conclusion

This study explored how written-translation MTI students in southeastern China subjectively organized enjoyment, anxiety, and boredom in translation learning. Using Q methodology, it identified two related but distinguishable emotion profiles. The mastery-oriented profile was characterized by achievement enjoyment associated with standards, personal expression, practical application, revision, and demanding tasks. The feedback-sensitive profile also valued achievement and practical application, but connected enjoyment more closely with task relevance, teacher feedback, classroom atmosphere, and instructional support. Interpreted through control-value theory, these findings suggest that, within this group of written-translation MTI students, emotions were shaped not simply by whether students valued translation learning, but by how they appraised their control over open-ended and professionally oriented translation tasks. Boredom did not constitute a separate profile in this study, but appeared as a secondary and situational emotion within profiles primarily differentiated by enjoyment, perceived competence, feedback, and task-related pressure.

### 6.2 Theoretical and pedagogical implications

Theoretically, this study contributes to research on achievement emotions in foreign language and translation learning in three ways. To begin with, it moves beyond variable-centered accounts of separate emotions by showing how enjoyment, anxiety, and boredom can be organized into shared subjective profiles. Additionally, it extends the use of control-value theory to the under-researched setting of MTI translation learning, where students’ emotional experiences are shaped by professional standards, revision, evaluation, certification pressure, and task authenticity. Finally, it adds nuance to multiple-emotion research by showing that the three focal emotions did not contribute equally to the formation of shared emotion profiles. In this study, boredom was present but less profile-defining than enjoyment and anxiety, suggesting that the relative salience of different achievement emotions may depend on how learners position a task within broader systems of value and control.

Pedagogically, the findings suggest that translation teachers should adopt a profile-sensitive understanding of students’ emotional experiences. For mastery-oriented students, demanding texts, professional standards, revision, and alternative solutions may not necessarily reduce enjoyment; instead, they can become sources of motivation when students perceive them as controllable and worthwhile. Teachers may therefore design tasks that combine clear standards with space for personal interpretation, stylistic decision-making, and repeated refinement. Authentic or simulated translation projects can also help students connect classroom learning with professional practice, thereby strengthening the perceived value of translation learning.

For feedback-sensitive students, emotional support may need to be built more explicitly into task design and classroom evaluation. Feedback is not merely encouragement but helps make standards visible, confirms progress, and reduces uncertainty in open-ended translation tasks. Translation teachers should therefore provide timely, concrete, and text-specific feedback, especially when students work with difficult texts or unfamiliar subject matter. Feedback should explain not only what is problematic in a translation but also why certain choices are more appropriate and how students can improve their work. In addition, public criticism, excessive peer comparison, and grade-centered teaching may intensify anxiety among students who are sensitive to classroom evaluation. Teachers can reduce these risks by creating a classroom environment in which multiple translation solutions are discussed constructively, errors are treated as resources for learning, and peer comparison is redirected toward collaborative analysis rather than competition.

The limited differentiating role of boredom further suggests that reducing boredom in translation learning should not be limited to making classes more entertaining. More fundamentally, teachers need to help students maintain both value and control. Tasks should be meaningful, appropriately challenging, and connected with visible progress. Revision activities, for example, may become less tedious when students can see how successive changes improve accuracy, style, communicative adequacy, or professional acceptability. In this sense, emotion-sensitive translation pedagogy should aim not only to reduce negative emotions, but also to help students understand demanding translation work as a process of growth, judgment, and professional development.

### 6.3 Limitations and future directions

Three limitations should be acknowledged. First, the study was conducted with a specific group of Chinese MTI students specializing in written translation. In line with the purpose of Q methodology, the findings identify shared patterns of subjectivity rather than estimate how widely these profiles are distributed among translation learners. The two profiles should therefore be understood as interpretive profiles emerging from the present research context, rather than as fixed categories representing all MTI students. Future studies could examine whether similar profiles appear across different institutions, regions, language pairs, and stages of translation training.

Second, although the Q-sorts and post-sort comments were appropriate for identifying and interpreting shared emotion profiles, the comments were elicited mainly in relation to participants’ most extreme placements and therefore provided useful but limited contextual evidence. Future research could combine Q methodology with in-depth interviews, classroom observations, reflective journals, or longitudinal designs to examine how emotion profiles develop across specific translation tasks, feedback episodes, revision processes, assessment contexts, and technology-mediated translation practices, particularly AI-assisted translation and post-editing.

Another limitation concerns the use of control-value theory. The study did not directly measure control or value appraisals; instead, these constructs were used as an interpretive lens for making sense of the Q-sort patterns and post-sort comments. Future research could combine Q methodology with control-value appraisal scales, interviews, or longitudinal classroom data to examine the appraisal processes underlying translation-learning emotions more directly.

Despite these limitations, the study demonstrates the value of Q methodology for revealing how translation learners subjectively organize multiple achievement emotions. It also shows that control-value theory can be used not only to explain individual emotions, but also to interpret shared emotion profiles in specialized language-learning contexts.

## Data Availability

The raw data supporting the conclusions of this article will be made available by the authors, without undue reservation.
