# Supplementary material for: Mastery-oriented and feedback-sensitive emotion profiles among written-translation MTI students in southeastern China: a Q methodology study
Source: Front Psychol. 2026 Jul 28;17:1906693. doi: 10.3389/fpsyg.2026.1906693 (PMC13457199; doi:10.3389/fpsyg.2026.1906693)
Supplement: Supplementary file 1 [file Supplementary_file_1.pdf]

Appendix A. Q-set and statement coding

Table A1. Chinese and English Q-set statements, domains, and emotional orientations

Statement	Original Chinese wording	English translation	Domain	Emotional orientation
S1	当一篇翻译作业太难，我几乎看不懂原文时，我会非常紧张，觉得压力很大。	When a translation assignment is too difficult and I can barely understand the source text, I feel very nervous and under great pressure.	D1	Anxiety
S2	我喜欢翻译那些与现实生活或社会热点紧密相关的文本，这会让我感觉翻译是有意义的。	I enjoy translating texts closely related to real life or current social issues because it makes me feel that translation is meaningful.	D1	Enjoyment
S3	当翻译任务太简单、几乎不用动脑时，我会觉得是在浪费时间。	When a translation task is too simple and requires little thinking, I feel it is a waste of time.	D1	Boredom + Anxiety
S4	当完成的译文符合课程要求或行业标准、且自己也觉得满意时，我会产生强烈的成就感。	When my completed translation meets course requirements or industry standards and I am also satisfied with it, I feel a strong sense of achievement.	D1	Enjoyment
S5	当老师给我们客户的真实项目或“仿真项目”时，我会觉得既兴奋又有一点紧张。	When the teacher gives us real or simulated client projects, I feel both excited and a little nervous.	D1	Enjoyment + Anxiety
S6	在翻译中不断修改、推敲用词的过程，会让我感到有点枯燥。	The process of repeatedly revising and deliberating over word choices in translation feels somewhat tedious to me.	D1	Boredom
S7	当我能将翻译课程学习成果用于实践，我会感到踏实和有成就感。	When I can apply what I have learned in translation courses to practice, I feel grounded and gain a sense of accomplishment.	D1	Enjoyment
S8	翻译文学文本时，即使机器翻译得不错，但自己能译出带有个人理解和风格的版本，会让我感到格外有意义和愉悦。	When translating literary texts, even if machine translation produces a decent version, being able to produce my own version with personal understanding and style makes the task feel especially meaningful and joyful.	D1	Enjoyment
S9	翻译专业性强的非文学文本时，我不仅觉得词汇量不足，更感到缺乏相关领域知识的支撑，这会让我压力很大。	When translating highly specialized non-literary texts, I feel not only that my vocabulary is insufficient but also that I lack necessary domain knowledge, which puts me under great pressure.	D1	Anxiety
S10	如果老师上课大部分时间都在单向讲解，很少互动，我会觉得翻译课很难熬。	If the teacher spends most of the class time lecturing unidirectionally with little interaction, I find translation class hard to endure.	D2	Boredom
S11	老师在课堂上鼓励不同译文、允许犯错时，我会更愿意大胆尝试翻译。	When the teacher encourages different translation versions and allows mistakes, I feel more willing to try out my own translation choices.	D2	Enjoyment
S12	每次翻译课我都担心被老师点名回答问题或当场翻译。	I worry about being called on by the teacher to answer questions or translate on the spot in every translation class.	D2	Anxiety
S13	老师幽默、会讲翻译背后的故事时，我会更享受翻译课堂。	When the teacher is humorous and tells stories behind translation, I enjoy translation class more.	D2	Enjoyment
S14	如果老师经常当众批评学生的错误，我会越来越不敢在课堂上发言。	If the teacher often criticizes students’ mistakes in public, I become increasingly afraid to speak up in class.	D2	Anxiety
S15	当老师只关注分数，不关心我们的感受时，我会对翻译课逐渐失去兴趣。	When the teacher focuses only on grades and does not care about our feelings, I gradually lose interest in translation class.	D2	Boredom + Anxiety
S16	老师及时、具体地指出我译文中的优点，会让我对翻译学习更有信心。	When the teacher promptly and specifically points out the strengths in my translation, I feel more confident in translation learning.	D2	Enjoyment

S17	如果老师布置作业很多但讲解不够，我会觉得又累又烦。	If the teacher assigns a lot of translation tasks but provides insufficient explanation, I feel tired and annoyed.	D2	Boredom + Anxiety
S18	当老师开放讨论不同译文、组织小组辩论时，我会觉得翻译课气氛很活跃。	When the teacher opens up discussion of different translations and organizes group debates, I feel the translation class atmosphere is very active.	D2	Enjoyment
S19	当老师布置了小组合作翻译任务时，我一方面因为分到的任务量减少而感到开心，另一方面也会因为要与小组成员沟通分工和译文而感到有压力。	When the teacher assigns a group collaborative translation task, I am happy on the one hand because my share of work is reduced, but on the other hand I feel stressed about coordinating the division of work and translation choices with group members.	D2	Enjoyment + Anxiety
S20	如果组里只有一两个人在认真做，其他人都在“划水”，我会觉得这种小组翻译很无聊。	If only one or two members in the group are working seriously while others are free-riding, I find such group translation boring.	D3	Boredom
S21	当同学展现出更强的翻译能力时，我既佩服又会觉得有些压力。	When classmates demonstrate stronger translation proficiency, I admire them but also feel some pressure.	D3	Enjoyment + Anxiety
S22	当同学们普遍对翻译课不感兴趣时，我也会受影响，觉得上课很无聊。	When students in general are not interested in translation class, I am also affected and find the class boring.	D3	Boredom
S23	如果组内氛围轻松，同学之间互相支持，我会更愿意在翻译活动中多尝试。	If the group atmosphere is relaxed and classmates support each other, I become more willing to participate actively in translation activities.	D3	Enjoyment
S24	在课堂上当众展示译文时，我有时会因为同学的评价而感到尴尬或不安。	When presenting my translation in front of the class, I sometimes feel embarrassed or uneasy because of classmates’ comments.	D3	Anxiety
S25	一想到翻译成绩要算入总绩点，我在做翻译作业时就会变得很紧张。	The thought that translation grades will be included in my overall GPA makes me very nervous when doing translation assignments.	D4	Anxiety
S26	当我在翻译课程考试、竞赛或证书考试中取得好成绩时，我会对自己的翻译能力更有信心。	When I achieve good results in translation course exams, competitions, or certification tests, I become more confident in my translation proficiency.	D4	Enjoyment
S27	为了一个重要的证书考试或翻译比赛进行高强度学习时，我会感到既充实又紧张。	When studying intensively for an important certification exam or translation competition, I feel both fulfilled and nervous.	D4	Enjoyment + Anxiety
S28	和刚入学相比，我现在更能享受翻译带来的思考和表达的乐趣。	Compared with when I first entered the MTI program, I now take greater pleasure in the thinking and expression involved in translation.	D5	Enjoyment
S29	我常常怀疑自己是否适合做翻译，想到这一点就会感到焦虑。	I often doubt whether I am suitable for translation, and this thought makes me anxious.	D5	Anxiety
S30	我会把翻译看作自己未来可能长期从事的职业，这一想法让我既期待又紧张。	I see translation as a possible long-term career for myself, a thought that makes me both hopeful and nervous.	D5	Enjoyment + Anxiety
S31	看到机器翻译越来越强大，我会对翻译专业的前景感到不安。	Seeing machine translation become more and more powerful makes me feel uneasy about the future of the translation profession.	D5	Anxiety
S32	如果长时间看不到自己翻译水平的明显进步，我会对继续投入产生怀疑。	If I do not see obvious progress in my translation level over a long period, I begin to question whether I should keep investing effort.	D5	Boredom + Anxiety
S33	长时间进行翻译实践，我会感到疲惫甚至有点厌烦。	Practicing translation for a long time makes me feel tired and even somewhat bored.	D6	Boredom
S34	如果我必须同时完成翻译作业和其他课程任务，我会感到被压得喘不过气来。	If I have to complete translation assignments and other course tasks at the same time, I feel overwhelmed.	D6	Boredom + Anxiety

S35	在状态好的时候，我可以忘记时间，沉浸在翻译任务中。	When I am in good form, I can lose track of time and become immersed in the translation task.	D6	Enjoyment
S36	当课程安排合理、任务量与难度匹配时，我会对整门翻译课保持比较稳定的积极情绪。	When the course schedule is reasonable and the workload matches the difficulty level, I maintain relatively stable positive emotions throughout the translation course.	D6	Enjoyment

Note. D1 = translation tasks and texts; D2 = teachers and classroom pedagogy; D3 = peers and interaction; D4 = assessment, examinations and certificates; D5 = self-efficacy and professional identity; D6 = workload, energy and burnout.

Appendix B. Q-methodological results for the retained two-factor solution

Table B1. Factor arrays and statement status for the retained two-factor solution

Statement	Factor 1 Q-SV	Factor 1 z-score	Factor 2 Q-SV	Factor 2 z-score	Status	Sig.
**S1**	+1	0.444	+2	1.065	F2 distinguishing	$p < 0.01$
**S2**	+2	0.923	+3	1.460	F2 distinguishing	$p < 0.01$
**S3**	-3	-1.426	-4	-1.496	Consensus	n.s.
**S4**	+4	2.057	+4	1.629	F1 distinguishing	$p < 0.05$
**S5**	+2	0.793	0	-0.205	F1 distinguishing	$p < 0.01$
**S6**	-4	-1.578	-2	-1.046	F2 distinguishing	$p < 0.01$
**S7**	+3	1.704	+4	1.902	Consensus	n.s.
**S8**	+4	2.087	+1	0.776	F1 distinguishing	$p < 0.01$
**S9**	+1	0.262	+2	0.949	F2 distinguishing	$p < 0.01$
**S10**	-1	-0.662	-1	-0.363	Consensus	n.s.
**S11**	+3	1.426	+2	1.060	F1 distinguishing	$p < 0.05$
**S12**	-1	-0.520	0	0.220	F2 distinguishing	$p < 0.01$
**S13**	+1	0.594	+3	1.322	F2 distinguishing	$p < 0.01$
**S14**	0	-0.242	+1	0.607	F2 distinguishing	$p < 0.01$
**S15**	0	-0.029	+1	0.557	F2 distinguishing	$p < 0.01$
**S16**	+3	0.987	+3	1.434	F2 distinguishing	$p < 0.05$
**S17**	0	-0.052	+1	0.338	F2 distinguishing	$p < 0.05$
**S18**	+1	0.402	0	-0.021	F1 distinguishing	$p < 0.05$
**S19**	-2	-0.933	0	-0.174	F2 distinguishing	$p < 0.01$
**S20**	-1	-0.269	0	0.002	Consensus	n.s.
**S21**	0	-0.200	+2	0.972	F2 distinguishing	$p < 0.01$
**S22**	-2	-1.186	0	-0.188	F2 distinguishing	$p < 0.01$
**S23**	0	0.132	0	0.277	Consensus	n.s.
**S24**	-2	-0.810	-1	-0.310	F2 distinguishing	$p < 0.01$
**S25**	-3	-1.392	-1	-0.494	F2 distinguishing	$p < 0.01$
**S26**	+2	0.966	0	-0.124	F1 distinguishing	$p < 0.01$
**S27**	+1	0.689	-2	-0.977	F1 distinguishing	$p < 0.01$
**S28**	0	-0.219	-3	-1.338	F1 distinguishing	$p < 0.01$
**S29**	-4	-1.510	-1	-0.896	F2 distinguishing	$p < 0.01$
**S30**	-1	-0.475	-4	-1.776	F1 distinguishing	$p < 0.01$
**S31**	0	0.077	-2	-1.122	F1 distinguishing	$p < 0.01$
**S32**	-1	-0.514	-2	-0.938	F1 distinguishing	$p < 0.05$
**S33**	-3	-1.448	-1	-0.619	F2 distinguishing	$p < 0.01$
**S34**	-2	-0.968	+1	0.317	F2 distinguishing	$p < 0.01$
**S35**	+2	0.822	-3	-1.457	F1 distinguishing	$p < 0.01$
**S36**	0	0.065	-3	-1.342	F1 distinguishing	$p < 0.01$

Note. Q-SV = Q-sort value in the factor array; n.s. = not significant. Z-scores are reported to three decimal places. Statements are ordered by statement number, with full wording provided in Appendix A. Distinguishing status was identified by comparing z-scores between the two factors.

Table B2. Factor loadings for participants

Participant	Factor 1 loading	Factor 2 loading	Status
**P01**	**0.5871**	0.5556	Factor 1 defining sort
**P02**	0.3170	**0.7534**	Factor 2 defining sort
**P03**	0.2947	**0.3397**	Factor 2 defining sort
**P04**	0.4156	**0.5350**	Factor 2 defining sort
**P05**	**0.6715**	0.2484	Factor 1 defining sort
**P06**	-0.0954	**0.7633**	Factor 2 defining sort
**P07**	0.1295	**0.7134**	Factor 2 defining sort
**P08**	0.3321	**0.4423**	Factor 2 defining sort
**P09**	0.3060	**0.3932**	Factor 2 defining sort
**P10**	**0.5815**	0.3060	Factor 1 defining sort
**P11**	**0.6066**	0.2835	Factor 1 defining sort
**P12**	**0.6497**	0.0296	Factor 1 defining sort
**P13**	0.2188	**0.4278**	Factor 2 defining sort
**P14**	**0.4266**	0.1133	Factor 1 defining sort
**P15**	**0.5077**	0.1185	Factor 1 defining sort
**P16**	**0.5865**	0.5218	Factor 1 defining sort
**P17**	0.3603	**0.5783**	Factor 2 defining sort
**P18**	0.3305	**0.6814**	Factor 2 defining sort
**P19**	0.3768	**0.6327**	Factor 2 defining sort
**P20**	**0.5273**	0.4932	Factor 1 defining sort
**P21**	**0.7684**	0.4157	Factor 1 defining sort
**P22**	**0.6148**	-0.0138	Factor 1 defining sort
**P23**	**0.6178**	0.2988	Factor 1 defining sort
**P24**	0.3022	0.2857	Non-defining sort
**P25**	**0.7924**	0.2493	Factor 1 defining sort
**P26**	**0.8013**	0.3674	Factor 1 defining sort
**P27**	**0.6936**	0.1724	Factor 1 defining sort
**P28**	0.1043	**0.4079**	Factor 2 defining sort
**P29**	0.3991	**0.5460**	Factor 2 defining sort
**P30**	-0.0820	**0.8983**	Factor 2 defining sort

Note. Defining sorts were auto-flagged at $p < 0.05$ with the majority-of-common-variance requirement. No Q-sort was retained as confounded under these criteria; Q-sorts with secondary high loadings were assigned only when the majority-of-common-variance criterion was met.
